# 8-Hydroxy-2-Deoxyguanosine and 8-Iso-Prostaglandin F2α: Putative Biomarkers to assess Oxidative Stress Damage Following Robot-Assisted Radical Prostatectomy (RARP)

**DOI:** 10.3390/jcm11206102

**Published:** 2022-10-17

**Authors:** Alessandro Di Minno, Achille Aveta, Monica Gelzo, Lorella Tripodi, Savio Domenico Pandolfo, Felice Crocetto, Ciro Imbimbo, Giuseppe Castaldo

**Affiliations:** 1 Dipartimento di Farmacia, Università degli Studi di Napoli Federico II, 80131 Naples, Italy; 2 CEINGE-Biotecnologie Avanzate, 80131 Naples, Italy; 3 Dipartimento di Neuroscienze, Scienze Riproduttive e Odontostomatologiche, Università degli Studi di Napoli Federico II, 80131 Naples, Italy; 4 Dipartimento di Medicina Molecolare e Biotecnologie Mediche, Università degli Studi di Napoli Federico II, 80131 Naples, Italy

**Keywords:** prostate cancer, robot-assisted radical prostatectomy, oxidative stress, 8-hydroxy-2-deoxyguanosine (8-OHdG) 8-iso-prostaglandin F2α (8-IsoF2α) liquid chromatography–tandem mass spectrometry (LC–MS/MS)

## Abstract

Objective: Prostate cancer (PCa) is the most common type of cancer. Biomarkers help researchers to understand the mechanisms of disease and refine diagnostic panels. We measured urinary 8-hydroxy-2-deoxyguanosine (8-OHdG) and 8-iso-prostaglandin F2α (8-IsoF2α) to assess oxidative stress damage in PCa patients undergoing robot-assisted radical prostatectomy (RARP). Methods: Forty PCa patients were enrolled in the study. Urine was collected before (T0) and 3 months after the RARP procedure (T1). 8-OHdG and 8-IsoF2α were measured through liquid chromatography-tandem mass spectrometry. Sex- and age-matched healthy subjects served as controls (CTRL). Results: At T0, patients exhibited significantly higher levels of 8-OHdG than CTRL (*p* = 0.026). At T1, 23/40 patients who completed the 3-month follow-up showed levels of 8-OHdG that were significantly lower than at T0 (*p* = 0.042), and comparable to those of the CTRL subjects (*p* = 0.683). At T0, 8-Iso-PGF2α levels were significantly higher in PCa patients than in CTRL subjects (*p* = 0.0002). At T1, 8-Iso-PGF2α levels were significantly lower than at T0 (*p* < 0.001) and were comparable to those of CTRL patients (*p* = 0.087). Conclusions: A liquid chromatography-tandem mass spectrometry method reveals enhanced OHdG and 8-Iso-PGF2α in the urine of PCa patients. RARP normalizes such indices of oxidative stress. Large-sized sample studies and long-term follow-ups are now needed to validate these urinary biomarkers for use in the early prevention and successful treatment of PCa.

## 1. Introduction

Prostate cancer (PCa), the most common cancer and the second most common in terms of death after lung cancer, is a major public health issue in developed countries [1,2,3]. In addition to age, risk factors [4] associated with PCa development include family history, ethnicity, genetic factors, diet, and lifestyle [5]. Age at diagnosis is also a major determinant of the incidence of mortality in PCa [6]. Robot-assisted radical prostatectomy (RARP) is the most innovative treatment for the surgery of PCa. Compared to open radical prostatectomy, RARP has a lower risk of positive surgical margin and a higher likelihood of the preservation of continence in high-risk settings [7,8]. In contrast, the data available are inadequate to prove the superiority of this approach in terms of oncological outcomes.

Metabolomic studies [9] may help to refine the pathophysiology and identify diagnostic biomarkers [10,11]. The equilibrium between DNA damage and repair is a physiological process [12]. By affecting the activity of sulfhydryl (SH)-dependent enzymes, reactive oxygen species (ROS), e.g., hydroxyl radicals, superoxide anion, and hydrogen peroxides shift the oxidant/antioxidant balance; this triggers genomic DNA damage and lipid peroxidation and leads to the injury of normal tissues [13]. Similar to other age-related diseases, PCa is often characterized by enhanced oxidative stress and oxidative damage [14,15]. One significant role of oxidative damage has been documented in the early stages of prostate carcinogenesis [14,16], as well as in PCa progression [17]. As with aging, chronic inflammation [18] and ischemia [19] enhance ROS generation and DNA damage, and lead to changes in antioxidant enzyme activity, triggering angiogenesis and premalignant and malignant modifications to the prostate. In view of its lowest redox potential, guanine is highly susceptible to oxidation [12]. The stable urinary metabolite of this nucleobase, 8-hydroxy-2-deoxyguanosine (8-OHdG), is a key biomarker of in vivo oxidative DNA damage [20,21]. Changes in the levels of 8-OHdG are associated with PCa onset and progression [22]. Little is presently known as to 8-OHdG modifications following prostatectomy [14,23].

Isoprostanes are a family of prostaglandin isomers derived from polyunsaturated fatty acids through the free radical-catalyzed peroxidation of arachidonic acid (5). They are measured in biological fluids, e.g., plasma and urine. The chemically stable 8-iso-prostaglandin F2α (8-iso-PGF2α) is a major isoprostane and is a reliable biomarker of free radical-catalyzed peroxidation (oxidative stress) [24]. Research evidence is accumulating that urinary 8-iso-PGF2α could serve as a noninvasive biomarker for prostaglandin F2α (PGF2α), which is a reliable index of cyclooxygenase-catalyzed inflammation [25].

Hence, to identify those molecules acting as biomarkers of the radicality of treatment in patients with PCa [7], we have measured the urinary levels of 8-OHdG and 8-iso-PGF2α before and after RARP surgery.

## 2. Materials and Methods

### 2.1. Chemicals and Reagents

For this study, 8-OHdG was purchased from Sigma-Aldrich (St. Louis, MO, USA); 15N5-8-OHdG was obtained from Cambridge Isotope Laboratories, Inc. (Andover, MA, USA), and 8-iso-PGF2α and 8-iso-PGF2α-d4 were from Cayman Chemicals Co. (Ann Arbor, MI, USA). Amicon^®^ ultracentrifugal filters (Ultracel^®^-30 K) were purchased from Merck Millipore, Ltd. (Cork, Ireland), and Sep-Pak^®^ C18 solid-phase extraction (SPE) cartridges (3 cc, 500 mg) were purchased from Waters (Milford, MA, USA). Purified water was obtained from the Milli-Q^®^ Integral system (Merck Millipore Ltd., Cork, Ireland). All other chromatography-grade chemicals were obtained from Sigma-Aldrich.

### 2.2. Study Population

The study was approved by the Ethics Committee of Federico II University. Patients presenting at our Institution with prostate-specific antigen (PSA) elevations and/or specific findings upon rectal examination were studied. Forty consecutive patients for whom the decision to undergo RARP was based on a diagnosis of PCa, established by a pathological examination, were included in the present study. All subjects underwent a detailed physical examination by the same urologist. In addition to PSA values, information on patients’ age, body mass index (BMI), smoking status, alcohol consumption, LDL and HDL cholesterol, and triglycerides were collected prior to surgery. Patients with a known history of cardiac, kidney, liver, or endocrinological disease, smokers, and patients consuming alcohol, as well as those with a history of lipid-lowering or antioxidant drugs or vitamins, and those with the clinical or radiological suspicion of metastasis were excluded from the study. Twelve sex- and age-matched healthy individuals without PCa history and without a recent history of infection or drug use acted as the control (CTRL) group.

### 2.3. Sample Preparation

Urinary samples were collected from the CTRL group at the scheduled visit. To assess the effect of prostatectomy intervention on oxidative-induced DNA damage in PCa patients, urine samples were collected before (T0) and 3 months after (T1) prostatectomy. In both the CTRL and patient groups, urine specimens for 8-OHdG and 8-iso-PGF2α measurement were collected early in the morning, aliquoted in tubes, and stored at −80 °C until analysis.

### 2.4. 8-Hydroxy-2-Deoxyguanosine Measurement

A liquid chromatography–tandem mass spectrometry (LC–MS/MS) method was set up and validated to measure urinary 8-OHdG levels [26]. Briefly, frozen urine was thawed at room temperature and then heated at 37 °C for 10 min to redissolve possible analyte precipitates [27,28]. Samples were centrifuged at 1700× *g* for 10 min. Subsequently, 200 μL aliquots were diluted with 200 μL of 15N5-8-oxodG internal standard solution (final concentration 5 ng/mL) and filtered through a 30,000 NMWL (nominal molecular weight limit) centrifugal filter at 10,000 × *g* for 30 min. The filtrate was injected into the LC–MS/MS system. The analytical instrument was a 5500 QTrap linear ion-trap quadrupole mass spectrometer (AB Sciex, Milan, Italy) outfitted with an electrospray ionization (ESI) source operating in positive mode. Chromatographic separation was performed using a pentafluorophenyl Kinetex F5 100 Å analytical column (100 × 2.1 mm, Phenomenex, Torrance, CA, USA) packed with 2.6 μm of core-shell particles, maintained at 30 °C. The mobile phase was set at a flow rate of 0.25 mL/min, using ammonium acetate 10 mmol/L (solvent A) and ammonium acetate 10 mmol/L in acetonitrile/water at 50: 50  *v*/*v* (solvent B). The samples (10 μL) were eluted with a gradient of mobile phase during a total run time of 14 min. The selected reaction monitoring (SRM) was performed by monitoring the transitions of m/z 284.0 → m/z 168.1 (8-oxodG) and m/z 289.0 → m/z 173.0 (15N5-8-oxodG). The operating conditions for MS analysis were the following: spray voltage, 2200 V; capillary temperature, 280 °C; sheath gas, 25 UA; auxiliary gas, 10 UA. The values of this analyte were reasonably stable over time in individual healthy subjects.

### 2.5. 8-Iso-Prostaglandin f2α Measurement

For 8-iso-PGF2α, the processing of the collected urine has previously been reported [29]. Briefly, urinary 8-iso-PGF2α was detected in the urine after solid-phase extraction (SPE). One and one-half milliliters of urine were spiked with 150 µL of the deuterated internal standards solutions (8-iso-PGF2a-d4) and purified using an SPE cartridge. The eluate, obtained by ethyl acetate, was evaporated to dryness under a vacuum and reconstituted with 150 µL of H2O:CH3CN (90:10 *v/v*). Ten microliters of the reconstituted urine sample were injected onto a Hypersil Gold C18 column (100 × 2.1 mm, 3 µm) at 30 °C and analyzed in a 5500 QTrap linear ion trap quadrupole mass spectrometer (AB Sciex, Milan, Italy) equipped with electrospray ionization source (ESI) operated in negative-ion mode. The LC mobile phases were (A) methanol:2.5 mM ammonium acetate (3:97 *v/v*) and (B) MeOH:acetonitrile (3:97 *v/v*). The gradient (flow rate of 200 µL/min) was recorded as follows: t0 90% A, t9 60% A, t12 5% A, t19 5%, t20 90% A, t40 90% A. Previous data show that there is minimal day-to-day isoprostane variation within a single patient, and urine samples can, therefore, be taken at any point throughout a test date [10,12]. The values of this analyte were reasonably stable over time in individual healthy subjects.

### 2.6. Statistics

Numerical variables were expressed as mean and standard difference (SD), median and interquartile range, frequency, or percentage, where appropriate. A paired Student’s *t*-test was used for between-group comparisons, while inter-group analyses were performed using an unpaired *t*-test. Pearson’s rank correlation was used for associations. Here, *p*-values of < 0.05 were considered statistically significant. All the analyses were performed using SPSS version 27 (IBM SPSS Statistics, IBM Corp, Armonk, NY, USA).

## 3. Results

### 3.1. Population

The demographic and clinical features of the study group are reported in Table 1. The RARP patients were younger and had a lower BMI than the CTRL group. Two patients underwent hormonal treatment and four underwent radiation therapy following the T1 visit, due to a PSA increase. No other difference was found between the two populations.

### 3.2. 8-OHdG Levels in Patients Undergoing RARP Surgery

**Figure 1:**
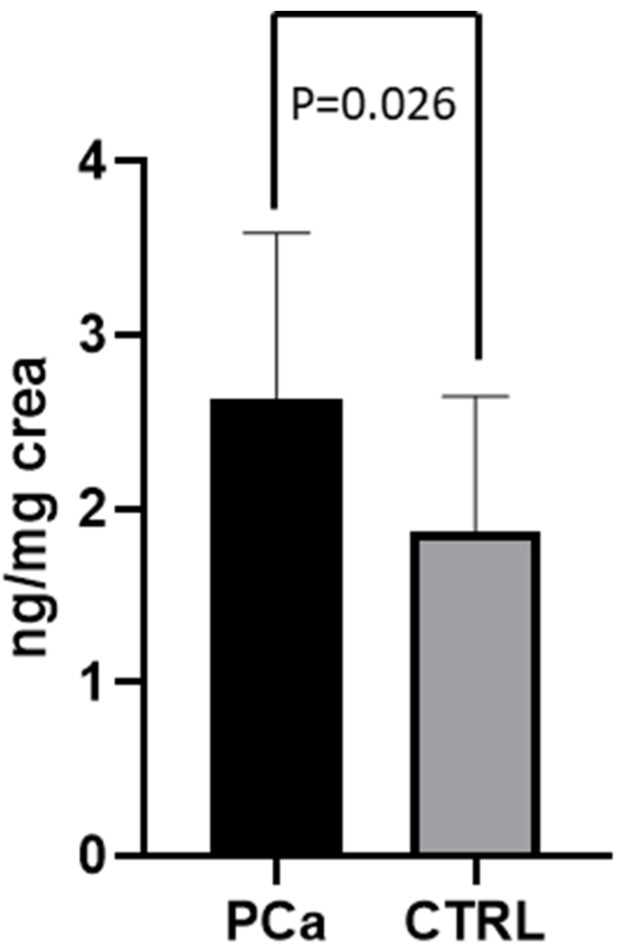
8-OHdG levels in PCa patients at T0 and CTRL. Levels of 8-OHdG measured in urine from PCa patients (at T0) and CTRL subjects. Data are represented as means  ±  SD.

**Figure 2:**
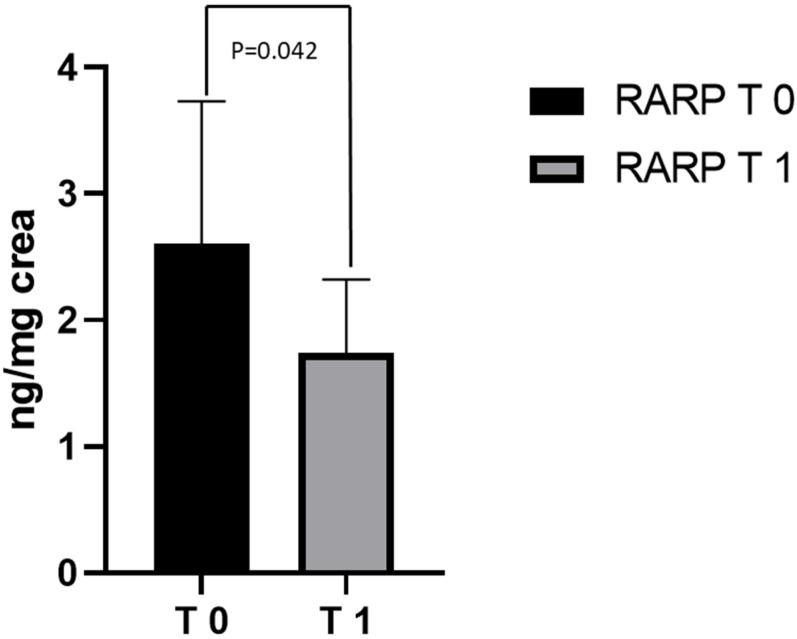
8-OHdG levels in RARP patients at T0 and T1. Levels of 8-OHdG measured in urine from RARP patients at T0 and T1. Data are represented as means  ±  SD.

**Figure 3:**
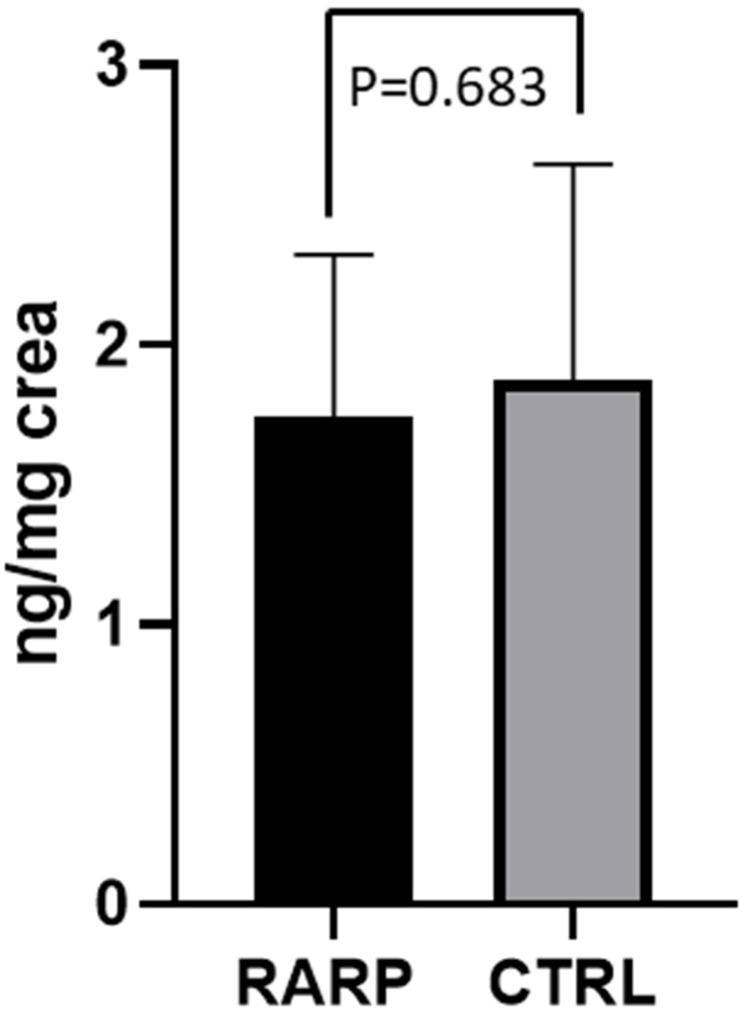
8-OHdG levels in RARP patients at T1 and CTRL. Levels of 8-OHdG measured in urine from RARP patients at T1 and CTRL subjects. Data are represented as means  ±  SD.

Before RARP (T0), the values of 8-OHdG (2.67 ±  0.96 ng/mg creatinine) in the 40 PCa patients that were examined were significantly higher than those of the CTRL group (1.87 ±  0.77 ng/mg creatinine) (*p* = 0.026 Figure 1). At T0, half of the patient population had metabolite excretion > 1 SD above the control mean. A significantly positive correlation between 8-OHdG levels and age (r = 0.495; *p* = 0.016) was observed over the whole population. In contrast, enhanced 8-OHdG was unrelated to cardiovascular risk factors and was independent of detectable atherosclerotic disease. Out of these 40 PCa patients, 23 underwent RARP in our hospital and completed the 3-month follow-up period. Compared to T0, a significant reduction in 8-OHdG levels was observed in the patients following the RARP procedure (2.60 ±  1.12 ng/mg creatinine at T0 vs. 1.74 ±  0.57 ng/mg creatinine at T1, respectively; *p* = 0.042; Figure 2). A 50% reduction in metabolite excretion was observed in all the patients in this setting. The 8-OHdG values following RARP were comparable to those observed in the CTRL group in this setting (1.74 ±  0.57 ng/mg creatinine and 1.87 ±  0.77 ng/mg creatinine, respectively; *p* = 0.683; Figure 3). No differences were found between the 2 patients who underwent hormonal therapy or the 4 patients who received radiation therapy and those who did not receive any adjuvant treatment (1.9 ± 0.74 in those receiving hormonal therapy vs. 1.67 ± 0.55 in those who did not, *p* = 0.57; 1.1 ± 1.62 in those receiving radiation therapy vs. 1.81 ± 0.57 who received no radiation therapy, *p* = 0.27, respectively).

### 3.3. 8-Iso-PGF2α Levels in Patients Undergoing RARP Surgery

**Figure 4:**
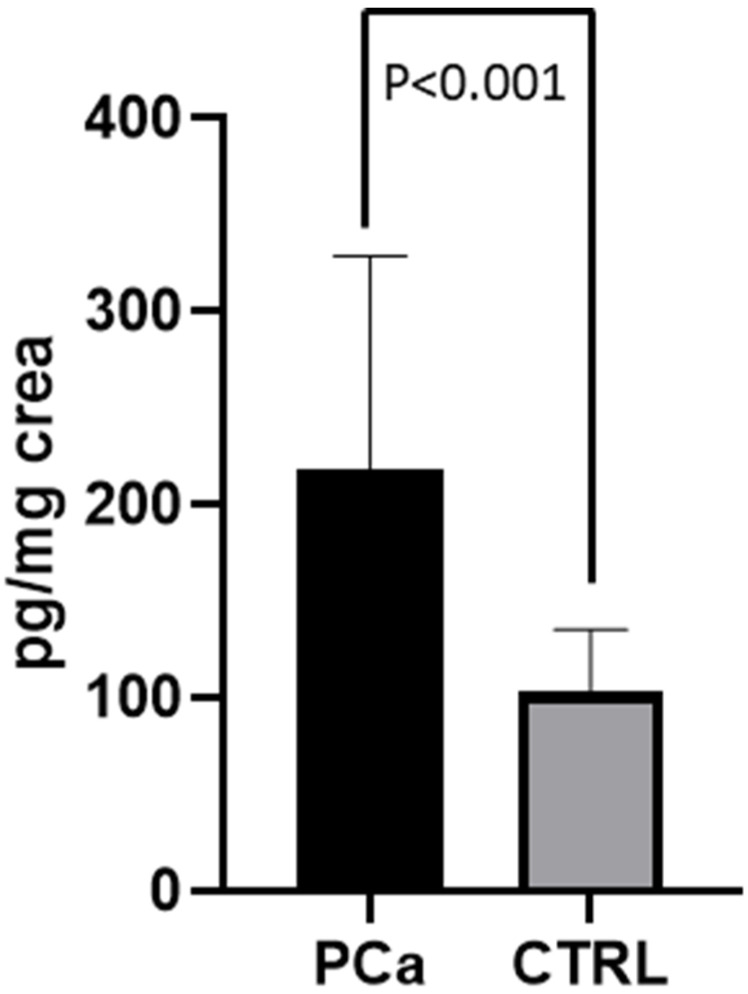
8-Iso-PGF2α levels in PCa patients at T0 and CTRL. Levels of 8-Iso-PGF2α measured in urine from PCa patients (at T0) and CTRL subjects. Data are represented as means  ±  SD.

**Figure 5:**
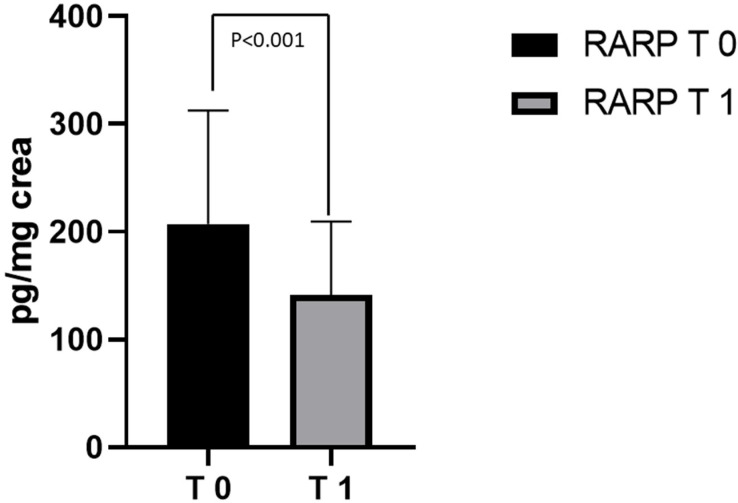
8-Iso-PGF2α levels in RARP patients at T0 and T1. Levels of 8-Iso-PGF2α measured in urine from RARP patients at T0 and T1. Data are represented as means  ±  SD.

**Figure 6:**
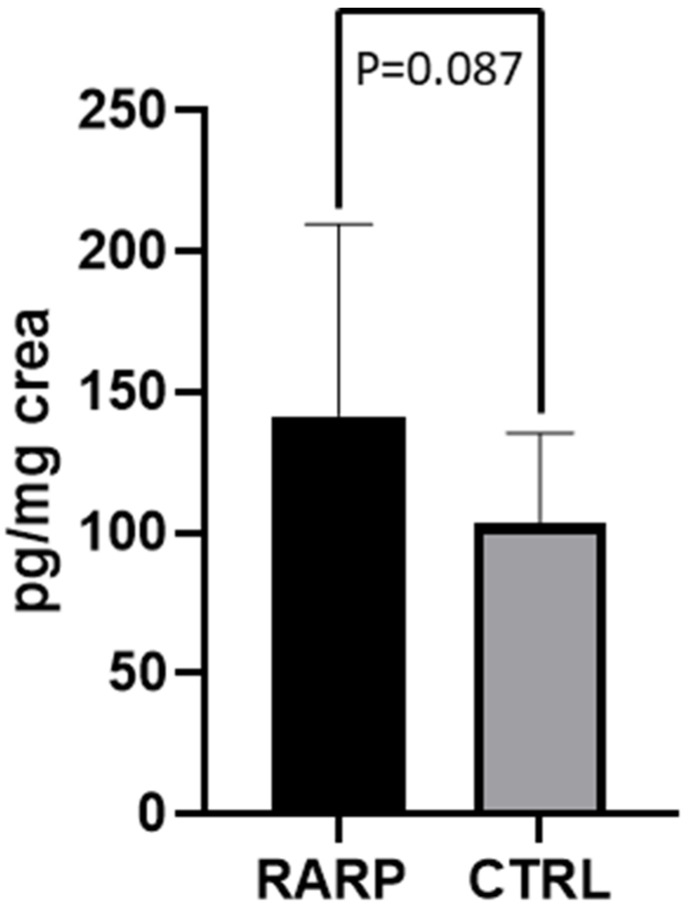
8-Iso-PGF2α levels in RARP patients at T1 and CTRL. Levels of 8-Iso-PGF2α measured in urine from RARP patients at T1 and CTRL subjects. Data are represented as means  ±  SD.

The values of 8-iso-PGF2α measured in the 40 PCa patients before RARP (T0) were twice as high as those of the CTRL subjects (218.11 ±  110.82 pg/mg creatinine; 103.38 ±  32.18 ng/mg creatinine, respectively; *p* = 0.0002). Metabolite excretion > 1 SD above the control mean was found in ≈2/3 of the PCa patient population examined (Figure 4). In the patients (*n* = 23) who had completed the 3-month follow-up period following the surgical procedure (T1), an almost 50% reduction in 8-Iso-PGF2α values was observed, compared to T0 (207.11 ±  105.42 pg/mg creatinine; 141.61 ±  67.98 ng/mg creatinine respectively; *p* < 0.001; Figure 5). At T1, no significant difference was found between 8-Iso-PGF2α excretion in PCa patients and the CTRL group (141.61 ±  67.98 pg/mg creatinine; 103.38 ±  32.18 ng/mg creatinine respectively; *p* = 0.087 Figure 6). No differences were found between the 2 patients who underwent hormonal therapy or the 4 patients who received radiation therapy and those who did not receive any adjuvant treatment (98.5 ± 38.77 in those receiving hormonal therapy vs. 150.70 ± 69.98 in those who did not, *p* = 0.17; 121.10 ± 36.41 in those receiving radiation therapy vs. 143.59 ± 70.51 who received no radiation therapy, *p* = 0.66).

## 4. Discussion

In this paper, we report that in the urine samples of patients with PCa, two major indices of oxidative stress damage, i.e., 8-OHdG and 8-iso-PGF2α, are consistently and significantly higher than in the CTRL group. We also show that successful surgery corrects such enhanced oxidative indices in PCa patients. Indeed, 3 months after RARP, the 8-OHdG and 8-IsoF2α levels were comparable to those found in the CTRL group.

ROS are a heterogeneous group of substances containing highly reactive ions and molecules derived from oxygen, such as the superoxide anion (O^2–^), hydroxyl radical (OH_), hydrogen peroxides (H_2_O_2_), and singlet oxygen (O^2–^). ROS are detoxified by antioxidants such as vitamins C and E and enzymes such as paraoxonases (PONs) [30]. ROS trigger DNA damage, changes in transcription and replication, the initiation of signaling transduction pathways, and genomic instability. These conditions are all relevant in cancer development. Abnormal PONs activity levels in patients with colorectal cancer will return to normal levels after surgery [31]. Similar findings have been reported in patients with papillary thyroid cancer, prior to and following total thyroidectomy [32].

Oxidative stress affects the development and progression of PCa [33], while accumulated DNA damage increases the risk of prostate cancer [34]. 8-OHdG is largely used to measure oxidative DNA damage [19]. As many as 42% of men aged 55–80 years exhibit prostate DNA damage, as assessed by 8-OHdG levels in tissue and urine samples [35]. Lipid peroxidation is a free-radical reaction that involves the oxidative conversion of polyunsaturated fatty acids to malondialdehyde (MDA) or other lipid hydroperoxide products, e.g., 4-hydroxy-2-nonenal (4HNE) [36]. MDA is highly mutagenic, while 4HNE is a very toxic by-product of lipid peroxidation. Conjugation with intracellular glutathione peroxidase (GSH) and glutathione S-transferase (GST) enables their detoxification [37]. Abnormal lipid peroxidation triggers changes in the cellular antioxidant system, particularly in the glutathione metabolizing enzymes, during cancer progression. Our data on 8-IsoF2α are, at least in part, in keeping with reports showing higher lipid peroxidation in prostate cancer cells than in benign tissue [38]. They also confirm and extend the information that the levels of lipid peroxides are abnormally high in prostate cancer patients [39]. As is the case for 8-OHdG, in this study, normalization of the levels of 8-Iso-PGF2α was detected after surgical excision of the malignancy.

The finding of normal levels of the markers of oxidative stress in patients three months after successful RARP suggests that surgical excision of the prostate was associated with the removal of a key source of an excess of free radicals. Despite the fact that the biomarkers studied in the present report lack tissue specificity [40], these data argue that 8-OHdG and 8-Iso-PGF2α measurements in urine can help to predict radicality (and maybe local recurrence) following PCa surgery. In this study, we used a highly specific and sensitive validated LC–MS/MS method to detect changes in oxidative stress markers in urine samples. Consistent and reproducible results were obtained with respect to the measurement of OHdG and 8-Iso-PGF2α. The methods employed may easily be duplicated in other laboratories. The finding that measurements of urinary 8-OHdG and 8-and Iso-PGF2α levels help to monitor changes in DNA damage and lipid peroxidation in PCa patients (and in high-risk subjects) should now be considered.

The sample size and the control of confounding variables (e.g., age) are obvious shortcomings of the present study. Furthermore, only half of the population had completed the 3-month follow-up. No differences in the levels of these analytes were found between patients who underwent hormonal or radiation therapies and those who did not receive adjuvant treatment(s) (8-iso-PGF2α: *p* = 0.17 and 0.66; 8-OHdG: *p* = 0.57 and 0.27, respectively). However, of the subjects studied, only 2 received radiation therapy and only 4 received hormonal therapy. Future work in larger groups of PCa patients will be aimed at analyzing the differences in these analytes related to adjuvant treatments.

The 8-OHdG and 8-iso-PGF2α values are abnormal in the present study because they are involved in a variety of pathological conditions where the common markers are oxidative stress, ischemia, and/or inflammation. PSA, the most common marker of PCa, is also affected by inflammatory states. The expression levels of 8-OHdG clearly differentiate PCa from benign prostatic hyperplasia. Indeed, compared to the specimens of benign prostatic hyperplasia, higher levels of 8-OHdG are found in specimens of PCa [19]. Nevertheless, these molecules, where the levels are normalized following the RARP procedure, as in the present setting, could in no way be used as biomarkers of disease relapse/recurrence. The presumed minimally invasive nature of robotic surgery in patients undergoing this procedure needs to be confirmed [41,42]. Large-scale trials are needed to address this concept and document the superiority of one surgical procedure vs. the other [43,44]. The results of these large-scale studies are an obvious prerequisite to employing these oxidative stress variables as good markers for staging or for predicting prognosis in PCa patients.

## 5. Conclusions

Significant, reproducible links between the enhanced indices of oxidative stress and PCa, and between radical prostate cancer removal and the normalization of such indices are revealed by measuring 8-OHdG and 8-Iso-PGF2α in the urine of patients with prostate cancer. Despite the fact that such biomarkers lack tissue and diagnostic specificity, our results argue for the measurement of 8-OHdG and of 8-Iso-PGF2α in urine before and after surgery as a technique to help predict radicality (and perhaps local recurrence) following PCa surgery, while large-sized sample studies and longer-term follow-ups are now needed to validate these urinary biomarkers in the pursuit of the early prevention and successful treatment of prostate cancer.

## Figures and Tables

**Table 1:** Characteristics of the RARP patients and control subjects.

Overall Population Characteristics at Baseline		
Variables	RARP (*n* = 40)	CTRL (*n* = 12)
**Demographic characteristics**		
Age, years	66.75 ± 6.61	69.25 ± 3.81
BMI, kg/m^2^	27.14 ± 2.91	28.35 ± 2.56
**Diagnosis**		
*Gleason Score*		
≤6 no. (%)	5 (12.5)	0 (0)
7 no. (%)	3 (7.5)	0 (0)
8–10 no. (%)	32 (80)	0 (0)
t-*score*		
1 no. (%)	0 (0)	0 (0)
2 no. (%)	29 (72.5)	0 (0)
3 no. (%)	11(27.5)	0 (0)
*PI-RADS*		
1–2 no. (%)	2 (5)	0 (0)
3 no. (%)	2 (5)	0 (0)
4 no. (%)	28(70)	0 (0)
5 no. (%)	7 (17.5)	0 (0)

Values are expressed as means ± SD or as *n* (% of total).

## Data Availability

Data available on request due to restrictions.
